# Complete Genome Sequences of Two Lytic Phages of Salmonella enterica Isolated from Wastewater in Ecuador

**DOI:** 10.1128/mra.01048-22

**Published:** 2023-01-18

**Authors:** Manuela Parra, Rosa de los Ángeles Bayas-Rea, Teresa Guerrero, Stuart Torres, Giuseppe D’Auria, Mariana Reyes-Prieto, Sonia Zapata

**Affiliations:** a Instituto de Microbiología, Colegio de Ciencias Biológicas y Ambientales, Universidad San Francisco de Quito, Quito, Ecuador; b Servicio de Secuenciación y Bioinformática, Fundación para el Fomento de la Investigación Sanitaria y Biomédica, Centro de Investigación Biomédica en Red en Epidemiología y Salud Pública, Valencia, Spain; Portland State University

## Abstract

Salmonella enterica is one of the most common causes of foodborne diseases. Bacteriophages provide an option to reduce the presence of Salmonella. Here, we describe the isolation of two lytic Salmonella bacteriophages. The complete genomes were annotated and show similarity to that of the lytic phage NBSal001, in the *Drexlerviridae* family.

## ANNOUNCEMENT

Salmonella enterica is one of the most common causes of foodborne diseases. Poultry meat and derivatives are the main sources of human infections. In Latin America, the prevalence of nontyphoid Salmonella (NTS) has increased, as well as the antimicrobial resistance (AMR). The use of bacteriophages for the control of Salmonella enterica colonization in the poultry industry is an alternative to reduce this prevalence. We report the complete genome sequences of two lytic Salmonella bacteriophages that are related to NBSal001, which was described previously; for this reason, we named Salmonella phages F115 and F61 NBSal001-like (1).

The lytic phages were isolated from wastewater collected on 12 February 2019 from the Machangara River (0°15′3.5″S, 78°31′28.0″W), which is the most contaminated river in Quito, the capital of Ecuador. We performed an enrichment of phages from a 45-mL river water sample using Salmonella enterica serovars Infantis and Typhimurium as hosts (2). We carried out 10-fold serial dilutions of phage stock solution to 10^−9^. Phages were purified from single plaque isolates using the double agar overlay assay described by Brown (2) with some modifications (brain heart infusion broth was used instead of Trypticase soy broth, and we supplemented the medium with Ca^2+^ instead of Mg^2+^).

We carried out five rounds of single plaque isolation for each phage (F115 and F61). The titer for each phage was 1 × 10^8^ PFU/ml. We used a Salmonella enterica serovar Infantis strain isolated from a poultry farm and Salmonella enterica serovar Typhimurium strain ATCC 14028 as hosts for the isolation of F115 and F61, respectively. We extracted DNA from 2 × 10^9^ PFU using a commercial kit (PureLink; Invitrogen) with the addition of RNase and DNase (3).

The library preparation for sequencing was performed as described in the Nextera DNA Flex library preparation protocol; the sequencing assay was performed using the Illumina MiSeq platform with the Nano 2 × 250-bp standard sequencing kit. With the resulting raw data, read trimming and quality assessment were performed with fastp v0.23.1 (4), and a report was generated using MultiQC v1.11 (5). The sequencing assay produced 228,241 and 314,602 reads for F115 and F61, respectively. The contig assembly was performed using MEGAHIT through SqueezeMeta v1.4.0 (6). Assembly resulted in 90 and 49 contigs for the F115 and F61 phages, respectively. In both cases, the resulting contigs included a single largest contig with high coverage, while the remaining contigs were very short and with low coverage. This finding was indicative of a complete phage genome (7). With BLASTn (8), phage NBSal001 (GenBank accession number MN994500) was assigned as the reference genome for our sequence queries because of its identity level (Table 1). To facilitate the comparison between genomes, the assembled contigs were reordered based on the direction and position of the NBSal001 genome (GenBank accession number MN994500) using a script by Wang et al. (mummer_direction.py) (9). Genome annotation was performed using RASTtk v1.073 (10–12). Default parameters were used for all software unless otherwise specified.

**TABLE 1:** Characteristics of phages F115 and F61

Parameter	Finding for phage:	
	F115	F61
GenBank accession no.	OP292673	OP292674
No. of reads (thousands)	228.2	314.6
Genome length (bp)	48,162	46,635
Coverage (×)	252	252
No. of genes	77	76
No. of genes with predicted functions	52	51
GC content (%)	42.3	42.2
% identity with NBSal001 (GenBank accession no. MN994500)	99.72	99.71
% identity with FSL_SP126 (GenBank accession no. NC_042066)	93.09	93.10

We report two Salmonella bacteriophage genomes, with genome coverage of 252× and genome lengths of 48,162 bp and 46,635 bp. The average GC content is 42%. Phage F115 has 77 predicted coding genes, while phage F61 has 76. At the nucleotide level, these phages share more than 99% identity with NBSal001 (GenBank accession number MN994500) and 93.01% with phage FSL_SP126 (GenBank accession number NC_042066), both in the *Drexlerviridae* family (Table 1).

### Data availability.

The annotated genome assemblies have been deposited in GenBank under the accession numbers OP292673 (F115) and OP292674 (F61). The raw data are available in the NCBI Sequence Read Archive (SRA) under BioProject accession number PRJNA893638, SRA accession numbers SRS15587974 and SRS15587975, and BioSample accession numbers SAMN31429972 and SAMN31429973.
